# Safety and efficacy of rebamipide compared to artificial tears for the treatment of dry eye: a systematic review and meta-analysis

**DOI:** 10.1186/s12886-025-04146-0

**Published:** 2025-05-27

**Authors:** Ching-Wen Chiu, Ka-Wai Tam, I-Chan Lin

**Affiliations:** 1 School of Medicine, College of Medicine, Taipei Medical University, Taipei, Taiwan; 2 Department of Medical Education, Taipei Veterans General Hospital, Taipei, 112201 Taiwan; 3 Division of General Surgery, Department of Surgery, Shuang Ho Hospital, Taipei Medical University, New Taipei City, Taiwan; 4 Division of General Surgery, Department of Surgery, School of Medicine, College of Medicine, Taipei Medical University, Taipei, Taiwan; 5 Cochrane Taiwan, Taipei Medical University, Taipei, Taiwan; 6 Department of Ophthalmology, School of Medicine, College of Medicine, Taipei Medical University, Taipei, Taiwan; 7 Department of Ophthalmology, Wan Fang Hospital, Taipei Medical University, Taipei, Taiwan

**Keywords:** Dry eye disease, Rebamipide, Systematic review, Meta-analysis, Tear breakup time

## Abstract

**Background:**

Rebamipide (RBM) is a novel drug that increases mucin secretion on the ocular surface. Nevertheless, the therapeutic efficacy of topical RBM for dry eye disease (DED) treatment remains inconclusive. Accordingly, we conducted a systematic review and meta-analysis of randomized controlled trials (RCTs) investigating the effectiveness of RBM for DED treatment.

**Methods:**

We searched the PubMed, Embase, and Cochrane Library databases for eligible RCTs. The primary outcome was posttreatment changes in tear breakup time (TBUT). We also assessed changes in the values of Schirmer’s test (Sch), corneal fluorescein staining scores, and DED-related symptom scores.

**Results:**

We retrieved 12 RCTs with 1368 eyes published during 2012–2023. The results demonstrated that compared with artificial tears, 2% RBM significantly increased the TBUT [standard mean difference (SMD) = 1.42, 95% confidence interval (CI) = 0.20 to 2.64]. Moreover, 2% RBM led to more significant improvements in overall DED-related symptom scores than did artificial tears (SMD = − 1.61, 95% CI = − 2.61 to − 0.61). The differences in the adverse events between the 2% RBM and artificial tears groups were nonsignificant (SMD = 1.23, 95% CI = 0.62 to 2.44).

**Conclusion:**

Topical RBM ophthalmic suspension is a safe and effective treatment for DED patients. Compared to artificial tears, 2% RBM improved TBUT and subjective symptoms of DED. It may be considered as the first-line treatment option for short- TBUT dry eye patients.

**Supplementary Information:**

The online version contains supplementary material available at 10.1186/s12886-025-04146-0.

## Background

Dry eye disease (DED) is a multifactorial disorder characterized by increased tear evaporation or insufficient tear production, leading to ocular discomfort and visual impairment [1]. The pathogenesis of DED includes increased tear film osmolarity as well as ocular surface and lacrimal gland inflammation. The prevalence of DED has increased from 5%–34% in 2003 to 5%–50% in 2017 [2–4]. Age, sex, and Asian ethnicity are among the consistently reported risk factors for DED [4, 5]. The clinical symptoms and economic burden engendered by DED have negative effects on patients’ quality of life, work productivity, and psychological status [6].

DED treatment modalities are prescribed on the basis of symptom severity, and they include artificial tears (the mainstay of DED therapy), anti-inflammatory agents (e.g., cyclosporine A), tear fluid and mucin secretion promoting agents (e.g., diquafosol), mucin secretagogue (e.g. rebamipide (RBM)), and antibiotics (e.g., macrolides and tetracycline) [5]. RBM ophthalmic solution (e.g., MUCOSTA Ophthalmic Suspension UD 2% from Otsuka Pharmaceutical, Tokyo, Japan) is a novel quinolinone derivative that improves tear film stability by increasing membrane-associated mucin levels [7–9] and protects corneal epithelial cells from the TNF-α–induced disruption of barrier function [10, 11].

The effectiveness of RBM for DED treatment has been demonstrated in multiple clinical studies, including randomized controlled trials (RCTs) and cohort studies [12–19]. For example, Kinoshita et al. compared the treatment efficacy of RBM and artificial tears against DED and revealed that compared with artificial tears, RBM improved both objective signs and subjective symptoms of DED [14, 15]. Several studies have demonstrated that RBM improved the tear breakup time (TBUT) and ocular surface condition in patients with DED who underwent ocular surgery [13, 16, 18].

Although several studies have assessed the efficacy of RBM against DED, their findings are limited by their small sample sizes. Accordingly, to address these limitations, we conducted this systematic review and meta-analysis to comprehensively review and analyze RCTs investigating the therapeutic effects of RBM on the objective signs and subjective symptoms of DED.

## Methods

### Selection criteria

We included RCTs investigating the effects of topical RBM in patients with DED. We included RCTs that met the following criteria: (1) enrolling patients with clinical symptoms or signs of DED, (2) including at least 10 patients, and (3) providing clear reports of posttreatment outcomes. We excluded RCTs that met at least one of the following criteria: (1) including animals and (2) reporting outcomes other than clinical symptoms or signs.

### Search strategy and study selection

This systematic review was registered on PROSPERO, the online international prospective register of systematic reviews of the National Institute for Health Research. (CRD42023483246).

In September 2023, we comprehensively searched the PubMed, Embase, and Cochrane Library databases for eligible full-text RCTs by using the following Medical Subject Headings terms and Boolean operators: *((keratoconjunctivitis sicca) OR ((((((Dry eye) OR (Dry eye disease)) OR (DED)) OR (dry eye disorder)) OR (dry eye syndrome)) OR (DES))) AND ((rebamipide) OR (RBM))*. In the PubMed search, we used the “Related articles” feature to broaden the search. No language or publication date restrictions were applied to the literature search. We also searched the reference sections of relevant articles and contacted known experts in the field. Finally, we searched for unpublished studies in the ClinicalTrials.gov registry (http://clinicaltrials.gov/).

### Data extraction

Two reviewers (CHC and KWT) independently extracted the following details from the RCTs: population characteristics, inclusion and exclusion criteria, node evaluation procedures, and statistical definition of outcomes. The independently recorded decisions of the two reviewers were compared, and any disagreements were resolved through evaluation by a third reviewer (ICL).

### Methodological quality assessment

The methodological quality of the included RCTs was assessed using RoB 2.0, a revised tool for assessing risk of bias in RCTs [20]. Two reviewers (CHC and KWT) independently evaluated bias in five domains for each outcome: (1) randomization process, (2) deviations from intended interventions, (3) missing outcome data, (4) measurement of the outcome, and (5) selection of the reported result. A third reviewer (ICL) resolved any disagreements between the two reviewers. Each RCT was assigned an overall risk-of-bias rating according to the highest risk of bias calculated for it in any of the aforementioned domains.

### Outcome assessment

Efficacy was evaluated primarily with an objective measure and secondarily with objective and subjective measures. The primary objective endpoint was the TBUT, and the secondary objective endpoints were the value of Schirmer’s test (Sch), corneal fluorescein staining (CFS) scores, and lissamine green conjunctival staining (LGCS) scores. In general, a TBUT of > 10 s and an Sch value of > 10 mm in 5 min are considered normal [1, 21, 22]. Moreover, CFS and LGCS scores are generally derived using an Oxford grading scale ranging from 0 (*absent*) to 5 (*severe*) [23]. The secondary subjective endpoints included DED-related ocular symptoms (i.e., foreign-body sensation, dryness, photophobia, eye pain, and blurred vision) and patients’ overall treatment impression. These symptoms were assessed using a scoring system ranging from 0 to 4, where 0 represented the absence of symptoms, and 4 indicated very severe symptoms or symptoms that occurred continuously [13, 15]. Higher scores corresponded to more severe symptoms experienced by the patient. If the included studies utilized different point scales to assess subjective dry eye symptoms, we would convert them proportionally to a 4-point scale to facilitate a comprehensive analysis.

### Statistical analysis

The changes in baseline and endpoints for our meta-analysis. For RCTs reporting baseline and endpoint measurements, we calculated changes in scores by using the method recommended in the *Cochrane Handbook*; through this method, we assumed a correlation of 0.5 between the baseline and endpoint measurements [24]. All data were entered into and analyzed on Review Manager (version 5.3.5; Cochrane Collaboration, Oxford, UK). We conducted our meta-analysis in accordance with the Preferred Reporting Items for Systematic Reviews and Meta-Analyses guidelines [25].

Continuous outcome data were analyzed using the standard mean difference (SMD). The precision of an effect size is expressed through a 95% confidence interval (CI). A *P* value of < 0.05 was considered to indicate statistical significance. Statistical heterogeneity was assessed using the *I*^*2*^ test; *I*^*2*^ was used to quantify the proportion of the total outcome variability attributable to the heterogeneity among the included studies. To facilitate reporting, we tentatively divided heterogeneity levels into the following categories: low (*I*^*2*^ = 25%–50%), moderate (*I*^*2*^ = 51%–75%), and high (*I*^*2*^ = 76%–100%) heterogeneity [26].

## Results

### Study characteristics

**Fig. 1:**
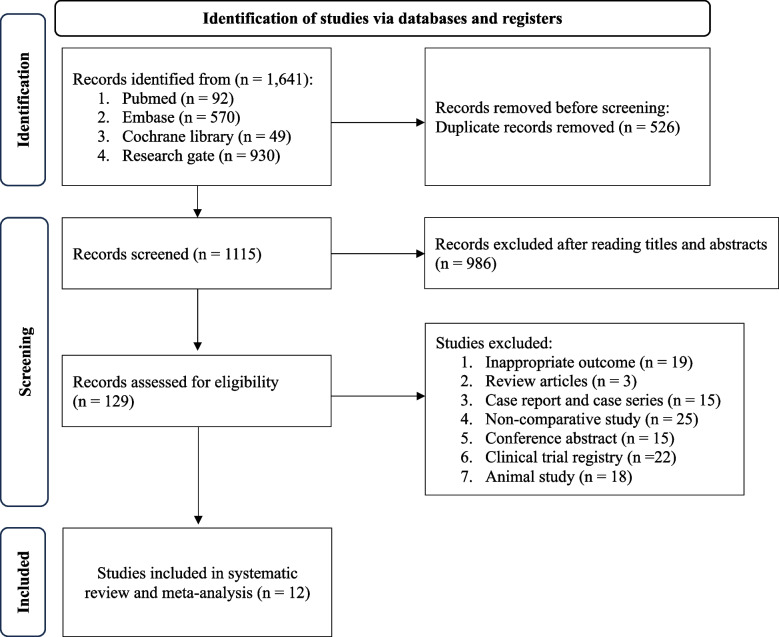
Flowchart of study selection process

Figure 1 illustrates a flowchart of the study screening and selection process. Our initial search yielded 1,641 results, of which 526 were duplicates and were thus removed. After screening the titles and abstracts of the remaining 1,115 studies, we determined that 986 studies were ineligible and thus excluded them. The excluded studies included those investigating the physiological effects of RBM on DED, reviewing the management of DED, or examining the therapeutic effects of RBM on autoimmune disease-associated dry eye. Next, we retrieved the full text of the remaining 129 studies for further review. Of these remaining studies, 117 were excluded from our final analysis for the following reasons: 19 considered irrelevant outcomes, 3 were review articles, 15 were case reports or case series, 25 were noncomparative studies, 15 were conference abstracts, 22 were ongoing clinical protocols, and 18 were animal studies. The remaining 12 eligible studies [12–16, 27–33] were included in our analysis; their characteristics are listed in Table 1. These 12 studies were published between 2012 and 2023 and had patient sample sizes ranging from 25 to 308, including a total of 1,368 eyes.

**Table 1:** Baseline characteristics of included studies

Study name (year)	Inclusion criteria	No. of patients (% male)	Age (years), mean ± SD	TBUT (sec), mean ± SD	Follow up period	Washout/run-in period	Intervention
Eom et al. (2023) [12]	Symptoms > 6 mo; CFS ≥ 4; Sch ≤ 10 mm/5 min	R_1_: 146 (80.6) R_2_: 74 (86.5) C: 74 (83.8)	R_1_: 43.3 ± 13.6 R_2_: 44.2 ± 14.8 C: 44.0 ± 14.4	R_1_: 3.68 ± 1.84 R_2_: 3.69 ± 1.78 C: 3.67 ± 2.96	4, 8, 12 wk	2 wk	R_1_: RBM 2% (20 mg/mL) R_2_: RBM 1% (10 mg/mL) C: control
Gandhi et al. (2023) [27]	DED-related symptoms ≥ 1; Sch score ≤ 10 mm; TBUT ≤ 10 s	R_1_: 30 R_2_: 30 C_1_: 30 C_2_: 30 Total: 120 (35)	NR	R_1_: 5.56 R_2_: 5.4 C_1_: 5.73 C_2_: 5.38	2, 4, 12 wk	NR	R_1_: RBM 2% R_2_: RBM 2% + CsA 0.05% C_1_: HA 0.1% C_2_: HA 0.1% + CsA 0.05%
Jain et al. (2023) [28]	DED-related symptoms ≥ 1; abnormal signs ≥ 1^a^	R: 80 eyes (42.5) C: 80 eyes (40)	R: 61–75 y: 12 pts > 75 y: 4 pts C: 61–75 y: 6 pts > 75 y: 4 pts	R: Moderate: 42 eyes C: Moderate: 41 eyes	2, 6, 12 wk	NR	R: RBM 2% four times daily C: CMC 0.5%
Rajguru et al. (2021) [30]	DED-related symptoms ≥ 1; abnormal signs ≥ 1	R: 40 C: 40	NR	NR	12 wk	NR	R: RBM 2% C: CMC 0.5%
Sharma et al. (2021) [31]	DED-related symptoms ≥ 1; Sch ≤ 10 mm; TBUT ≤ 10 s	R: 65 (52.3) C: 65 (49.2)	R: 66.55 ± 11.739 C: 65.20 ± 11.071	R: 8.03 ± 1.25 C: 8.38 ± 1.208	2, 4, 6 wk	NR	R: RBM 2% C: HA 0.1%
Teshigawara et al. (2021) [33]	DED-related symptoms ≥ 1; TBUT ≤ 5 s	R: 28 eyes C: 30 eyes	R: 66.57 ± 12.36 C: 66.07 ± 12.36	R: 2.73 ± 1.26 C: 2.61 ± 1.07	4 wk	NR	R: RBM 2% C: artificial tear (Mytear ophthalmic solution) four times daily
Patel et al. (2018) [29]	Score of > 2 for 1 or more DED-related ocular symptoms; Sch ≤ 5 mm/5 min; TBUT ≤ 5 s; BCVA ≥ 6/36	R: 50 C: 50 Total: 100 (65)	Total: 29.8 (18–54)^b^	R (L’t eye): 7^c^ C (R’t eye): 7.3^c^ R (L’t eye): 7^c^ C (R’t eye): 7.3^c^	2, 4, 8 wk	NR	R: RBM 2% C: CMC 0.5%
Kobashi et al. (2017) [16]	Corneal disease undergoing PK; mild to moderate dry eye; used artificial tear or other eye drops	R: 20 (55) C: 20 (50)	R: 72.4 ± 13.7 C: 68.3 ± 17.9	R: 3.3 ± 1.4 C: 3.9 ± 1.5	2, 4 wk	NR	R: RBM 2% four times a day C: DQS 3% tetrasodium ophthalmic solution four times a day
Shimazaki et al. (2017) [32]	DEQS ≥ 4; workers using computer > 4 h/d; 20 < age < 60	R: 40 (29.4) C: 39 (30.3)	R: 41.5 ± 9.6 C: 38.2 ± 9.7	R: 3.20 ± 0.84 C: 3.45 ± 1.02	2, 4, 8 wk	NR	R: RBM 2% C: DQS 3% tetrasodium ophthalmic solution
Igarashi et al. (2015) [13, 34]	DED after corneal refractive surgery; Sch ≤ 5 mm/5 min; TBUT ≤ 5 s	R: 15 (13.3) C: 10 (10)	R: 34.4 ± 10.8 C: 33.7 ± 10.8	R: 2.2 ± 0.7 C: 2.7 ± 0.8	4 wk	2 wk	R: RBM 2% four times daily C: artificial tears (preservative free) four times daily
Kinoshita et al. (2013) [15]	DED not fully alleviated; symptoms > 20 mo	R: 93 (10.8) C: 95 (15.8)	R: ≥ 65 y: 38 pts C: ≥ 65 y: 36 pts	NR	2, 4, 6 wk, and last observation carried forward	2 wk	R: RBM 2% for 1 drop (50 µl) in each eye 4 times daily C: HA 0.1% for 1 drop in each eye 6 times daily
Kinoshita et al. (2012) [14]	DED not fully alleviated; symptoms > 20 mo	R_1_: 102 (15.7) R_2_: 103 (9.7) C: 103 (12.6)	R_1_: ≥ 65 y: 41 pts R_2_: ≥ 65 y: 33 pts C: ≥ 65 y: 41 pts	NR	2, 4, 6 wk, and last observation carried forward	2 wk	R_1_: RBM 2% for 1 drop (50 µl) in each eye 4 times daily R_2_: RBM 1% for 1 drop (50 µl) in each eye 4 times daily C: PBO for 1 drop in each eye 4 times daily

*Abbreviations*: *BCVA* best corrected visual acuity, *C* control, *CFS* corneal fluorescein staining, *CMC* carboxymethylcellulose, *d* day, *DE* dry eye, *DED* dry eye disease, *DEQS* dry Eye–Related Quality-of-Life Score Questionnaire, *DQS* diquafosol, *HA* sodium hyaluronate ophthalmic solution, *I* intervention, *mo* month, *NR* not reported, *PBO* placebo, *PK* penetrating keratoplasty, *RBM* rebamipide, *Sch* Schirmer scores, *SCL* soft contact lens, *TBUT* tear film break-up time, *TED* thyroid eye disease, *wk* week, *y* year

^a^Signs: Sch, TBUT, CFS or rose Bengal dye

^b^Mean (range)

^c^Mean

Supplementary Fig. 1 in additional file 1 presents a summary of the methodological quality (RoB 2.0) of the included studies. Four studies—namely those by Igarashi et al. [13], Kinoshita et al. 2012 [14], Kinoshita et al. 2013 [15], and Shimazaki et al. [32]—demonstrated a low risk of bias in all five risk domains. Furthermore, four studies—namely those by Gandhi et al. [27], Kobashi et al. [16], Sharma et al. [31], and Teshigawara et al. [33]—demonstrated a low risk of bias associated with deviations from intended interventions, missing outcome data, measurement of the outcome, and selection of the reported result; however, they were also associated with some concerns regarding bias due to the randomization process. The study by Rajguru et al. [30] demonstrated a low risk of bias associated with missing outcome data, in addition to being associated with some concerns regarding bias due to the randomization process, deviations from intended interventions, measurement of the outcome, and selection of the reported result. Bias due to the randomization process was commonly noted among the included RCTs, mainly because these RCTs reported insufficient information regarding allocation concealment.

### Changes in TBUT from baseline to posttreatment endpoints

**Fig. 2:**
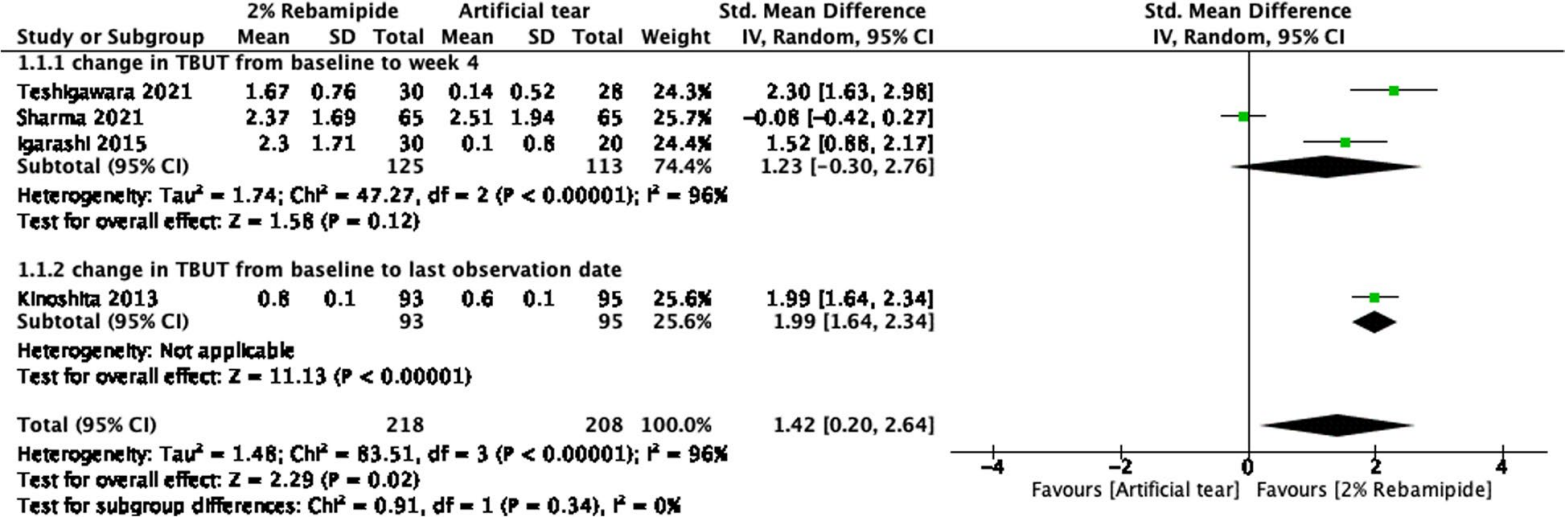
Forest plot of changes in TBUT after RBM and artificial tear administration. Values of change are reported with 95% Cis; *I*^2^ = 96%

Four RCTs compared such changes between 2% RBM and artificial tear groups [13, 15, 31, 33] (Fig. 2). Of these RCTs, three investigated these changes from baseline to posttreatment week 4 between the groups, whereas one investigates these changes from baseline to the final observation date. The pooled results demonstrated that compared with artificial tears, 2% RBM significantly increased the TBUT (SMD = 1.42, 95% CI = 0.20 to 2.64, *P* = 0.02).

Two RCTs compared changes in TBUT between 2% RBM and 1% RBM groups [12, 14] (Supplementary Fig. 2, Additional file 1). Overall, both 2% RBM and 1% RBM resulted in similar TBUT improvements (SMD = 0.01, 95% CI = − 0.20 to 0.22, *P* = 0.95). The two studies also compared changes in TBUT between 2% RBM and PBO groups (Supplementary Fig. 3, Additional file 1). Both 2% RBM and PBO resulted in similar TBUT improvements (SMD = 1.39, 95% CI = − 0.76 to 3.54, *P* = 0.21).

### Changes in the values of Schirmer test from baseline to posttreatment endpoints

Three RCTs compared changes in the value of Sch between 2% RBM and artificial tear groups [13, 15, 31] (Supplementary Fig. 4, Additional file 1). The pooled results revealed there were no differences on the changes of Sch values between 2% RBM and artificial tears (STD = − 0.62, 95% CI = − 2.03 to 0.78, *P* = 0.38). Two RCTs also compared changes in Sch scores between 2% RBM and 1% RBM groups [12, 14] (Supplementary Fig. 5, Additional file 1). There were no differences on the changes of Sch values between 2% RBM and 1% RBM (SMD = − 0.15, 95% CI = − 0.53 to 0.23, *P* = 0.45). These two RCTs also compared changes in Sch scores between 2% RBM and PBO groups (Supplementary Fig. 6, Additional file 1). There were no differences on the changes of Sch values between 2% RBM and PBO (SMD = 0.91, 95% CI = − 0.36 to 2.19, *p* = 0.16). However, the differences in Sch score improvement between the 2% RBM and 1% RBM or PBO groups were nonsignificant.

### Changes in corneal staining scores from baseline to posttreatment endpoints

Two RCTs compared changes in CFS scores from baseline to posttreatment endpoints between 2% or 1% topical RBM and PBO groups [12, 14]. There were no differences on the changes of CFS scores between 2% RBM and 1% RBM (SMD = − 0.48, 95% CI = − 1.50 to 0.55, *P* = 0.36; Supplementary Fig. 7, Additional file 1). There were no differences on the changes of CFS scores between 2% RBM and PMO group (SMD = − 0.48, 95% CI = − 13.92 to 4.32, *P* = 0.30; Supplementary Fig. 8, Additional file 1).

**Fig. 3:**
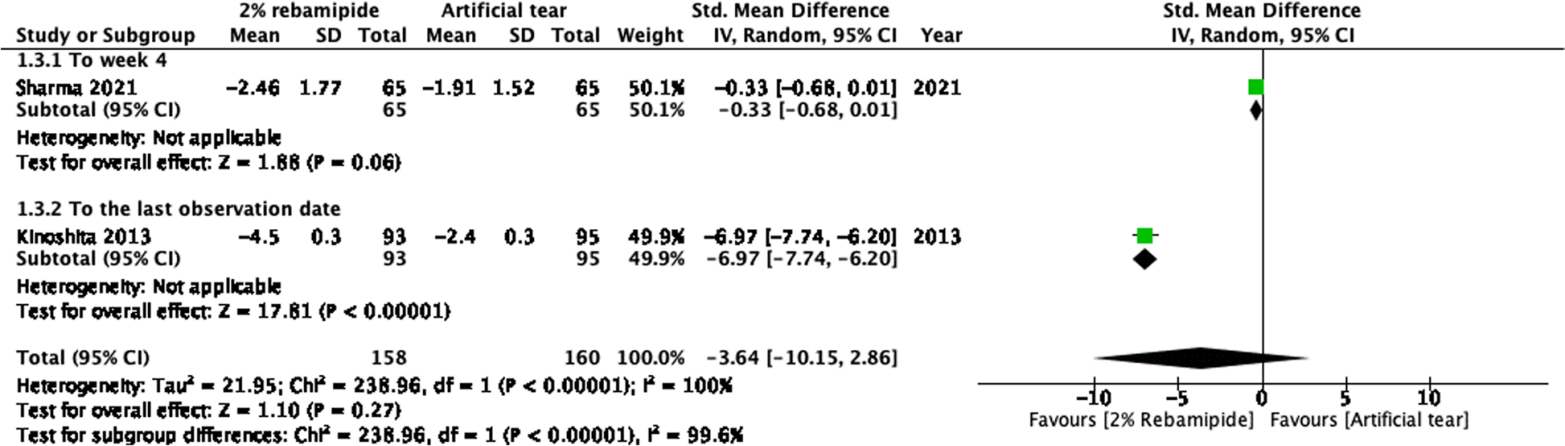
Forest plot of changes in LGCS scores after RBM and artificial tear administration. Values of change are reported with 95% Cis; *I*^2^ = 100%

Two RCTs compared changes in LGCS scores from baseline to posttreatment endpoints between 2% topical RBM and artificial tear groups [15, 31] (Fig. 3). A higher LGCS score typically indicates more severe DED. The pooled results revealed there were no differences on the changes of LGCS scores between 2% RBM and artificial tears (STD = − 3.64, 95% CI = − 10.15 to 2.86, *P* = 0.27).

### Changes in subjective symptoms from baseline to posttreatment endpoints

**Fig. 4:**
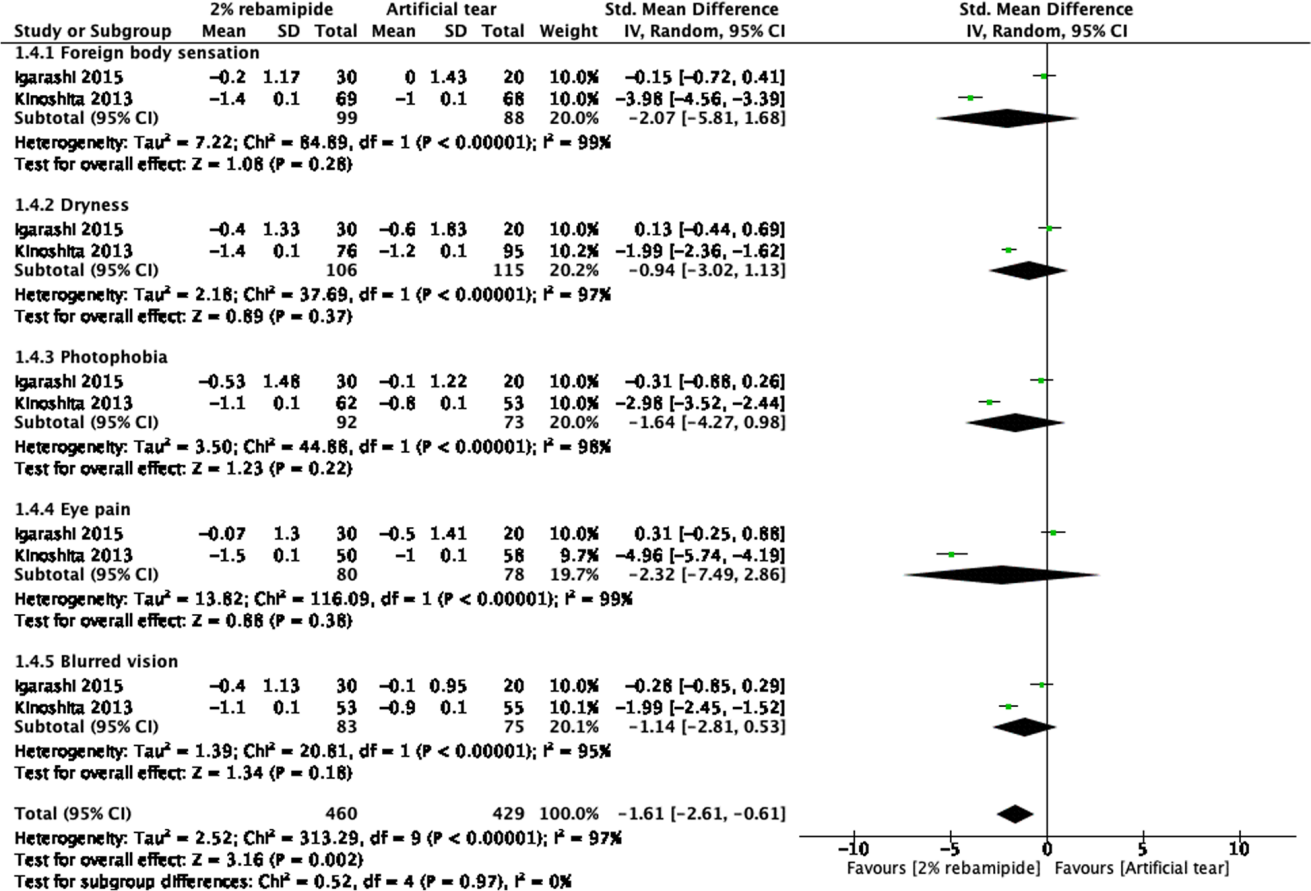
Forest plot of changes in DED-related symptom scores after RBM and artificial tear administration. Values of change are reported with 95% Cis; *I*^2^ = 97%

Two RCTs compared changes in DED-related ocular symptoms from baseline to posttreatment endpoints between 2% topical RBM and artificial tear groups [13, 16] (Fig. 4). A higher symptom score typically indicates more severe DED-related symptoms. Overall, 2% RBM engendered a more significant amelioration of DED-related symptoms than did artificial tears (SMD = − 1.61, 95% CI = − 2.61 to − 0.61, *P* = 0.002). Compared with artificial tears, 2% RBM did not ameliorate individual DED-related symptoms significantly; nevertheless, it ameliorated overall DED-related symptoms significantly.

### Adverse events

**Fig. 5:**
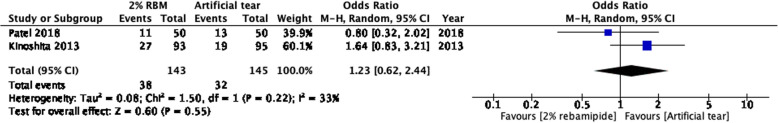
Forest plot of adverse events occurrence after RBM and artificial tear administration. Values of change are reported with 95% Cis; *I*^2^ = 33%

Figure 5 showed the adverse events between 2% topical RBM and artificial tear groups [15, 29]. Adverse events encompass both ocular and non-ocular occurrences. Non-ocular events reported consist of nasopharyngitis, decreased white blood cell count, dysgeusia, and headache. The differences in the adverse events between the 2% RBM and artificial tears groups were nonsignificant (SMD = 1.23, 95% CI = 0.62 to 2.44, *P* = 0.55).

## Discussion

Our study demonstrated that compared with artificial tears, 2% RBM increased the TBUT. In addition, compared with artificial tears, 2% RBM significantly ameliorated overall DED-related symptoms.

Although artificial tears are first-line treatment options for DED, they do not target factors underlying DED pathogenesis, such as mucin layer disruption [5, 35, 36]. Mucin layer disruption may lead to dysfunction in aqueous layer retention, further damaging the cornea and conjunctiva. Therefore, agents such as RBM, which increase mucin levels, represent an attractive therapeutic option for DED. Although numerous murine DED studies have assessed the treatment efficacy of RBM, the effectiveness of RBM in patients with DED remains relatively unclear. Accordingly, to fill this research gap, we conducted this systematic review and meta-analysis of all RCTs investigating the treatment efficacy of RBM in patients with DED.

Several studies have examined the effects of RBM on the TBUT in patients with DED. For example, Teshigawara et al. revealed that 2% RBM engendered significantly higher changes in TBUT than did artificial tears [18]. However, Sharma et al. demonstrated that although both 2% RBM and sodium hyaluronate induced improvements in TBUT, sodium hyaluronate engendered a greater improvement in TBUT [31]. Our meta-analysis results show that 2% RBM led to significant improvements in TBUT when compared with artificial tears. Moreover, we noted that the effectiveness of 2% RBM in improving TBUT was comparable to that of 1% RBM, with the difference between them being nonsignificant. Decreased TBUT are associated with the symptoms of blurring, pain, and photophobia [37]. In Asia, short TBUT DED are the most common type of DED, which is characterized by an unstable tear film [38–41]. For the short tear film breakup time-type DED, the goal of treatment is to stabilize the tear film, so the first option is to administer mucin-secreting eye drops [37]. Our study demonstrated RBM at both doses have greater improvements in TBUT than artificial tears. RBM may be considered as the first-line treatment option for those short-TBUT dry eye patients.

Sch is a diagnostic test that is commonly used to assess tear production and evaluate the presence of dry eye syndrome [21]. Studies on the effects of RBM on tear secretion in patients with DED have reported inconsistent results. For example, Kinoshita et al. reported that 2% RBM resulted in a significantly lower improvement in Sch scores at the final observation time point than did artificial tears or 1% RBM [15]. These results are in contrast with those of other included studies assessing improvements in Sch scores at the end of treatment [13, 31]. Our meta-analysis revealed that 2% RBM did not show significantly improve the Sch scores comparing to artificial tear treatment. Our findings support the argument of previous cohort studies that 2% RBM does not significantly improve tear production in patients with DED [34, 42, 43].

Although RBM did not significantly improve the CFS score in patients with dry eye disease compared to placebo, it demonstrated superior stabilizing ability, as evidenced by the two included studies [12, 14]. Take Eom et al. for example, they demonstrated that the 2% RBM group showed greater improvement in the CFS at 12 weeks from baseline than the placebo group [12]. The lack of significant improvement may be attributed to factors such as variations in the underlying causes of dry eye across different populations or ethnicities, as well as the limited sample size in the studies.

Previous trials have consistently noted dysgeusia as the most common adverse event associated with 2% RBM [15, 29]. Additionally, Kinoshita et al. observed higher occurrences of eye pruritis and decreased white blood cell count in patients receiving 2% RBM compared to artificial tears [15]. However, significant adverse events specifically linked to RBM treatment were not reported. Our study findings align with these previous observations, indicating no significant safety concerns related to RBM.

Because artificial tears currently constitute first-line treatment options in DED, comparing their effectiveness with that of RBM is essential. Sharma et al. reported that RBM led to a lower improvement in TBUT but greater improvements in Sch and ocular staining scores than did artificial tears. However, the differences between RBM and artificial tears in terms of improvements in these objective signs were nonsignificant [31]. Igarashi et al. revealed that RBM led to a significantly greater improvement in TBUT and nonsignificantly greater improvement in Sch scores than did artificial tears [13]. Several studies have also reported that RBM leads to significant improvements in TBUT, indicating that RBM is promising for improving tear film stability in patients with DED [17, 42, 43]. Our meta-analysis demonstrated that RBM resulted in significantly greater improvements in TBUT and overall DED-related symptoms. Therefore, RBM may be combined with artificial tears as a first-line treatment option, particularly in DED patients with tear film instability.

The studies included in our meta-analysis exhibited considerable heterogeneity because of various clinical factors. First, protocols for initiating topical RBM therapy, such as the washout period, were not standardized among the included studies; this thus increased the risk of drug overlap. Second, each artificial tear type demonstrated different levels of effectiveness in alleviating DED signs or symptoms. Third, DED severity in the patients differed among the included studies. However, despite this heterogeneity, our meta-analysis provides valuable insights, as the overall effect size remains robust and consistent across studies. The variability in study conditions reflects real-world clinical practice, enhancing the generalizability of our findings. Furthermore, the inclusion of diverse patient populations and treatment protocols strengthens the external validity of our results, making our study an important contribution to the existing body of evidence on RBM therapy for DED.

This study has some limitations. First, we included only RCTs, which may not reflect real-world outcomes. Second, variables such as rose bengal staining scores, DED-related quality-of-life scores, and ocular surface disease index values were not included; this could be attributed to the difficulty of defining subgroups or to the inadequacy of the data. Third, because of the inadequacy of relevant studies, we could not compare the effectiveness of RBM with that of each artificial tear type. Fourth, These questionnaires used in the included studies to assess DED symptoms lacked consistency in detail and validation. However, they still offer valuable insights by reflecting real-world clinical practice and the diverse assessment methods used in routine care. Future studies should consider using more reliable and validated tools, such as the Ocular Surface Disease Index (OSDI) or the Symptom Assessment in Dry Eye (SANDE), to enhance accuracy and comparability.

## Conclusions

In conclusion, topical REM ophthalmic suspension is a safe and effective treatment for DED patients. Our study revealed that 2% RBM increases the TBUT and ameliorates overall DED-related symptoms when compared with artificial tears. It may be considered as a first line treatment for short -TBUT DED patients. Further large-scale studies are warranted to investigate the real-world efficacy and safety of RBM in short-TBUT dry eye patients.

## Supplementary Information

Supplementary Material 1.

## Data Availability

No datasets were generated or analysed during the current study.

## Abbreviations

CFS: Corneal fluorescein staining
CI: Confidence interval
DED: Dry eye disease
LGCS: Lissamine green conjunctival staining
RCTs: Randomized controlled trials
RBM: Rebamipide
SMD: Standard mean difference
Sch: Schirmer’s test
TBUT: Tear breakup time
