# Supplementary material for: Safety and efficacy of rebamipide compared to artificial tears for the treatment of dry eye: a systematic review and meta-analysis
Source: BMC Ophthalmol. 2025 May 27;25:317. doi: 10.1186/s12886-025-04146-0 (PMC12107901; doi:10.1186/s12886-025-04146-0)
Supplement: Supplementary file 1 — Supplementary Material 1. [file 12886_2025_4146_MOESM1_ESM.docx]

**Supplementary Legends**

**Supplementary Figure 1.** Assessment (conducted using RoB 2.0) of quality of studies included in this meta-analysis

**Supplementary Figure 2.** Forest plot of changes in TBUT after 2% and 1% RBM administration. Values of change are reported with 95% Cis; *I*^2^ = 0%

**Supplementary Figure 3.** Forest plot of changes in TBUT after RBM and PBO administration. Values of change are reported with 95% Cis; *I*^2^ = 99%

**Supplementary Figure 4.** Forest plot of changes in Sch scores after RBM and artificial tear administration. Values of change are reported with 95% Cis; *I*^2^ = 97%

**Supplementary Figure 5.** Forest plot of changes in Sch scores after 2% and 1% RBM administration. Values of change are reported with 95% Cis; *I*^2^ = 69%

**Supplementary Figure 6.** Forest plot of changes in Sch scores after RBM and PBO administration. Values of change are reported with 95% Cis; *I*^2^ = 97%

**Supplementary Figure 7.** Forest plot of changes in CFS score after RBM and artificial tear administration. Values of change are reported with 95% Cis; *I*^2^ = 96%

**Supplementary Figure 8.** Forest plot of changes in CFS scores after RBM and PBO administration. Values of change are reported with 95% Cis; *I*^2^ = 100%


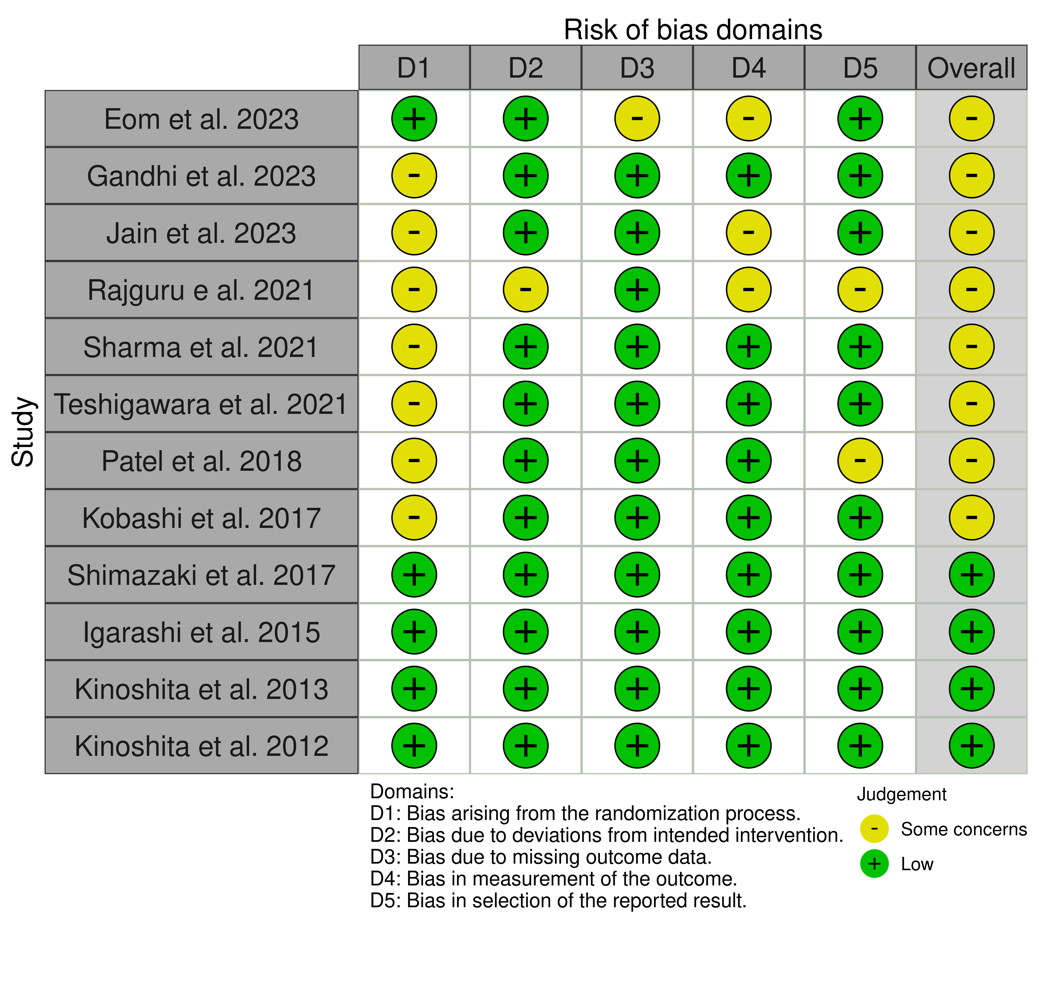

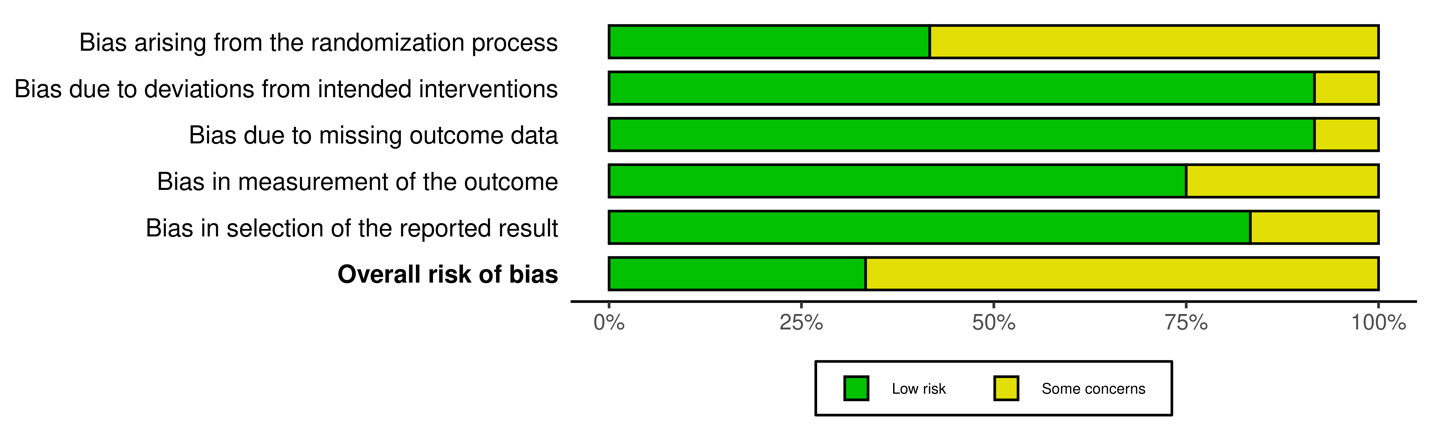


**Supplementary Figure 1.** ROB 2.0 for assessing the quality of included studies


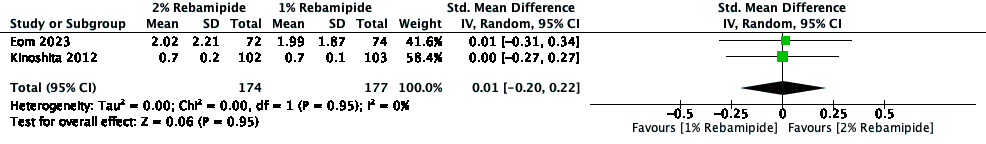


**Supplementary Figure 2.** Forest plot of changes in TBUT after 2% and 1% RBM administration


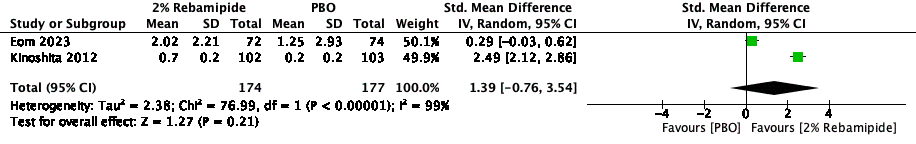


**Supplementary Figure 3.** Forest plot of changes in TBUT after RBM and PBO administration


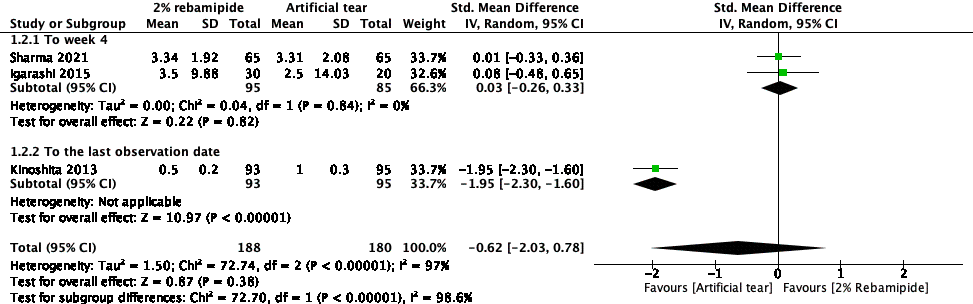


**Supplementary Figure 4.** Forest plot of changes in Sch scores after RBM and artificial tear administration


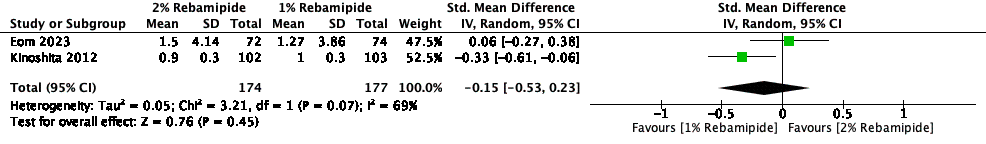


**Supplementary Figure 5.** Forest plot of changes in Sch scores after 2% and 1% RBM administration


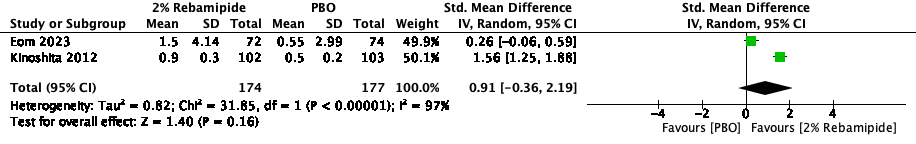


**Supplementary Figure 6.** Forest plot of changes in Sch scores after RBM and PBO administration


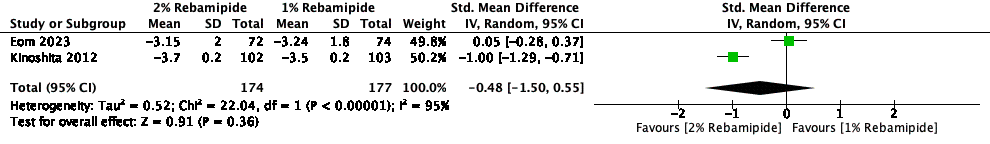


**Supplementary Figure 7.**Forest plot of changes in CFS score after RBM and artificial tear administration


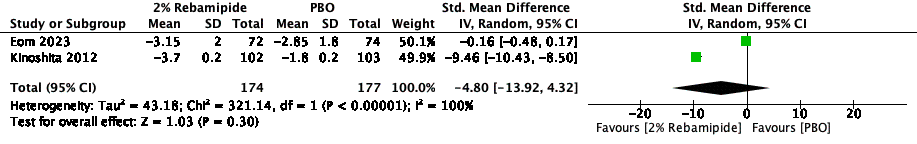


**Supplementary Figure 8.** Forest plot of changes in CFS scores after RBM and PBO administration
